# Automated Detection of Necrotizing Soft Tissue Infection Features by Computed Tomography

**DOI:** 10.3390/diagnostics15162030

**Published:** 2025-08-13

**Authors:** Heng-Yu Lin, Ming-Chuan Chiu, Tzu-Lun Kao, Chun-Chia Chen

**Affiliations:** 1 Division of Plastic Surgery, Department of Surgery, Chi Mei Medical Center, Tainan 710, Taiwan; hengyulin1222@gmail.com; 2 Department of Industrial Engineering and Engineering Management, National Tsing Hua University, Hsinchu 300044, Taiwan; mcchiu@ie.nthu.edu.tw (M.-C.C.); kaorichard1101@gapp.nthu.edu.tw (T.-L.K.); 3 School of Medicine, Chung Shan Medical University, Taichung City 40201, Taiwan

**Keywords:** necrotizing soft tissue infection, computed tomography, artificial intelligence, YOLOv10, object detection

## Abstract

**Background/Objectives**: To develop and evaluate an automated detection system for necrotizing soft tissue infection (NSTI) features on computed tomography (CT) images using the You Only Look Once version 10 (YOLOv10) model, aiming to improve diagnostic efficiency and surgical planning. **Methods**: This retrospective study included 31 patients with surgically confirmed NSTIs, spanning 2017–2023, from Chi Mei Medical Center, Taiwan. A total of 9001 CT images were annotated for four NSTI features: soft tissue ectopic gas, fluid accumulation, fascia edematous changes, and soft tissue non-enhancement. Model performance was evaluated using mean Average Precision (mAP), recall, and precision metrics. **Results**: The model achieved a mAP of 0.75, with recall and precision values of 0.74 and 0.72, respectively. Recall values for individual features were 0.76 for soft tissue ectopic gas, 0.66 for soft tissue non-enhancement, 0.92 for fascia edematous changes, and 0.68 for fluid accumulation. **Conclusions**: The YOLOv10-based system effectively detects four NSTI features on CT, including soft tissue ectopic gas, fluid accumulation, fascia edematous changes, and soft tissue non-enhancement.

## 1. Introduction

Necrotizing soft tissue infection (NSTI) is a life-threatening condition that without prompt surgical intervention can lead to rapid mortality [1]. Preliminary diagnosis primarily relies on patients’ clinical symptoms [2,3]. However, the early signs and manifestations of NSTIs closely resemble those of cellulitis or abscesses, and the relative rarity of NSTI further complicates accurate diagnosis. Studies indicate that 85–100% of patients are not diagnosed at their initial presentation [4,5,6]. Initially proposed to aid in distinguishing NSTIs from cellulitis using standard laboratory values, the Laboratory Risk Indicator for Necrotizing Fasciitis (LRINEC) score has been widely studied for its diagnostic utility. However, evidence from subsequent meta-analyses indicates that its sensitivity remains relatively low, typically between 40% and 68% [7]. This limited performance is likely due to the subtle or absent systemic immune activation in the early stages of NSTI, which can lead to falsely reassuring laboratory results.

Various imaging techniques are essential in early diagnosing NSTIs, with ultrasound, magnetic resonance imaging (MRI), and CT being commonly utilized [8]. Ultrasound can assist in excluding differential diagnoses such as deep vein thrombosis, detecting retained foreign objects, and guiding fluid aspiration when infection is suspected [9,10,11]. A key ultrasonographic sign—fluid accumulation greater than 2 mm in depth along the deep fascia—has been proposed as a supportive indicator for NSTI diagnosis [12]. Nonetheless, the effectiveness of ultrasound is heavily reliant on the skill and experience of the operator [13]. MRI, while historically regarded as the most accurate modality for evaluating soft tissue infections [14], is rarely employed in NSTI workups due to its lengthy acquisition time, which may delay urgent surgical treatment [15]. CT has demonstrated higher accuracy in diagnosing NSTIs and, in recent years, has been considered a viable diagnostic option when clinical signs are inconclusive [7]. A meta-analysis by Fernando et al. reported that CT imaging—when evaluating features such as fascial gas, edema, or enhancement—achieves a sensitivity ranging from 88.5% to 94.3% and a specificity between 76.6% and 93.3% [7]. Similarly, McGillicuddy et al. developed a CT-based scoring system that reached a sensitivity of 86.3%, specificity of 91.5%, and negative predictive value of 85.5%, underscoring the reliability of CT for NSTI detection [16]. CT not only facilitates NSTI diagnosis but also allows for precise identification of infection extent. However, this requires radiologists to spend significant time manually labeling large volumes of sectional images. As the number of images needing interpretation grows, the reduced time available for each assessment may increase the risk of errors [17].

AI has been shown to be beneficial in emergency radiology by assisting in the accelerated diagnosis of urgent conditions such as pulmonary embolism, small bowel obstruction, and acute stroke [18]. In addition to acute internal conditions, deep learning models have also been applied to skin and soft tissue disorders, such as the classification of pressure injuries in telemedicine settings [19], demonstrating the versatility of AI in image-based clinical decision support. At present, there is no published literature addressing the imaging analysis of soft tissue infections in emergency settings. The high utilization of CT in Taiwan’s emergency departments has allowed us to collect numerous CT images of NSTIs, enabling more in-depth research into this highly lethal infection.

The main obstacle in using artificial intelligence for NSTI diagnosis lies in the focus of existing research on detecting NSTI skin appearances alone. Das et al. used the YOLOv3 model to detect skin appearances for diagnosing NSTIs; however, they achieved only an Average Precision of 0.58 [20]. This may be since the most indicative sites for NSTIs are infections in the muscle fascia and deeper soft tissue structures rather than superficial skin appearance.

Our study aims to develop a model that uses artificial intelligence to automatically detect NSTI features in CT images. This model has the potential to enhance radiologists’ efficiency in diagnosing NSTIs. Additionally, more precise annotations could assist surgeons in creating more detailed preoperative plans, thereby reducing the likelihood of missed debridement areas.

## 2. Materials and Methods

### 2.1. Dataset

**Figure 1:**
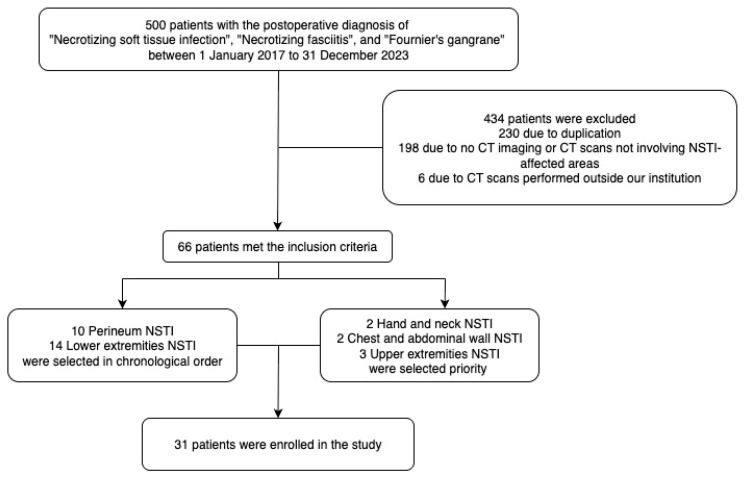
Patient selection flow chart.

In this study, cases were retrospectively collected from the Division of Plastic Surgery, Surgery Department at Chi Mei Medical Center from 1 January 2017, to 31 December 2023, with postoperative diagnoses containing keywords such as ‘Necrotizing fasciitis,’ ‘Necrotizing soft tissue infection,’ or ‘Fournier’s gangrene’. Exclusion criteria included the following: (1) patients without CT imaging; (2) CT images from patients outside of Chi Mei Medical Center; (3) CT scans not involving the area affected by NSTI; and (4) patients under 18 years of age. After screening 500 patients, a total of 66 cases with CT imaging were selected. The objective of this study aims to include images from various anatomical regions. Therefore, we prioritized images from the head and neck, chest and abdominal wall, and upper extremities, as NSTI cases in these regions are relatively rare. For the remaining images, newer records were prioritized. Ultimately, 31 cases with surgically confirmed NSTI diagnoses were selected for CT imaging (Figure 1).

### 2.2. Dataset Divisions

**Figure 2:**
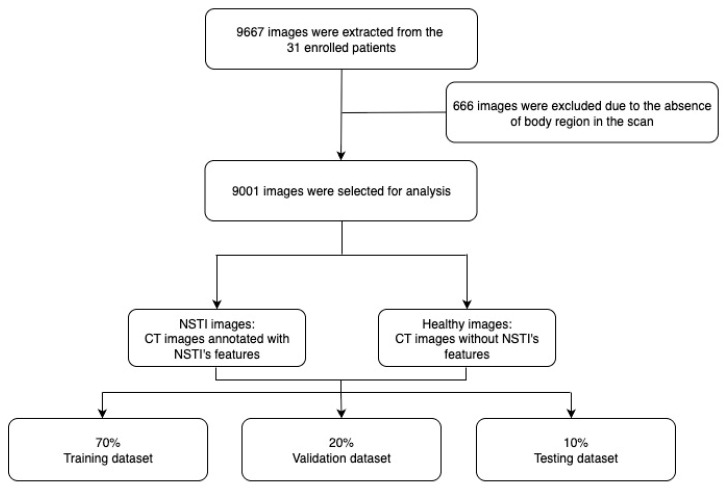
CT image processing with dataset division.

A total of 9667 CT images were extracted from 31 enrolled patients. Among them, 666 images were excluded due to the absence of relevant body regions in the scan, resulting in 9001 images retained for analysis. The dataset was then categorized into NSTI images—those annotated with NSTI features—and healthy images without such findings (Figure 2).

**Figure 3:**
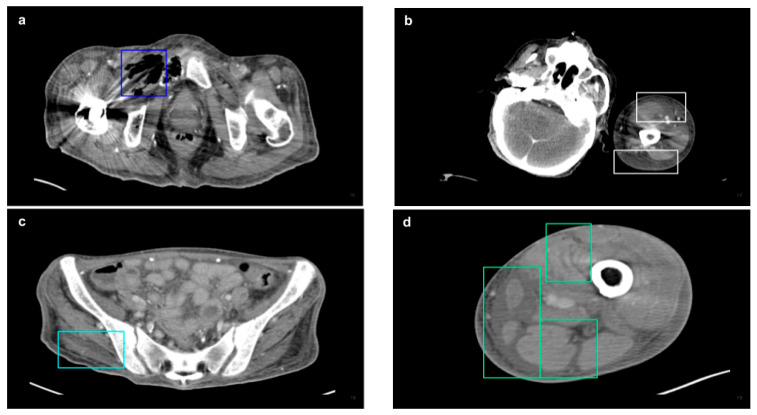
Annotated NSTI features. (**a**) The blue box highlights soft tissue ectopic gas accumulation within the right short external rotator muscles. (**b**) The white box indicates subcutaneous and fascial edematous changes in the anterior and posterior regions of the upper arm. (**c**) The light blue box delineates areas of soft tissue non-enhancement within part of the right gluteus maximus. (**d**) The green box captures fluid accumulation along the intramuscular plane, particularly within the medial compartment of the thigh.

Each image is annotated in a bounding box by experienced doctors, including the four main NSTI features: soft tissue ectopic gas [16,21,22,23], fluid accumulation [11,24,25,26], fascia edematous changes [11,23,27,28,29,30], and soft tissue non-enhancement [31,32] (Figure 3), while health images are not annotated. The annotation software used is the online Roboflow platform (https://roboflow.com/ (accessed on 1 August 2024)). After annotation, the dataset was divided into training, validation, and test sets. The dataset was divided into three parts: the training set, which comprised 70% of the total data and was used for model feature learning; the validation set, representing 20% of the data, which served as the optimization target during each iteration of model training to minimize loss; and the test set, constituting 10% of the data, which was used to evaluate the model’s performance after the training process was completed.

### 2.3. CT Acquisition and Analysis

Two different multidetector CT scanners were used for the CT examinations. Using a power injector, contrast-enhanced CT images were acquired by administering nonionic iodinated contrast material at a rate of 2–3 mL/s. Images were reconstructed axially, coronally and sagittally at 5-mm intervals, maintaining a uniform slice thickness of 5 mm.

### 2.4. Model Training

**Figure 4:**
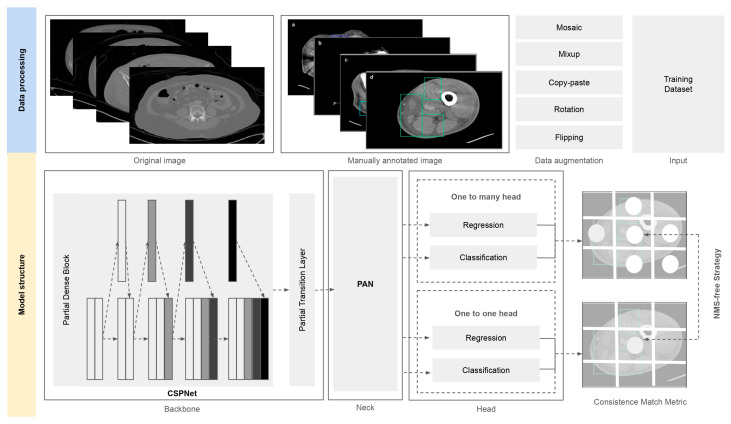
Overview of the YOLOv10-based detection framework for NSTI features on CT images. The model integrates a CSPNet backbone, PAN neck, and decoupled head with an NMS-free strategy.

The YOLOv10 model, an advanced real-time object detection framework known for its efficient detection speed and accuracy, was trained as shown in Figure 4. The backbone of YOLOv10 employs a Cross-Stage Partial Network (CSPNet) design, which enhances gradient flow and reduces computational redundancy through partial feature reuse. This structure improves the model’s ability to capture subtle and spatially sparse radiological patterns, such as soft tissue ectopic gas and fascia edematous changes, while maintaining real-time processing efficiency across high-resolution CT slices. YOLOv10 further incorporates Spatial-Channel Decoupled Downsampling (SCD) to improve resolution preservation during feature reduction, which is essential for detecting small or low-contrast abnormalities in medical images. The neck utilizes a Path Aggregation Network (PAN) to enhance multi-scale feature representation. PAN combines bottom-up and top-down pathways to fuse semantic information from deeper layers with spatially rich features from shallower layers. In the context of NSTI detection, this allows the model to maintain sensitivity to both fine-grained local features (e.g., fluid accumulation in the subfascial layer) and broader contextual cues (e.g., asymmetric soft tissue thickening across compartments). YOLOv10’s PAN implementation includes lightweight lateral modules to reduce channel redundancy and promote faster inference without sacrificing detail fidelity. The detection head in YOLOv10 introduces a decoupled structure with classification and localization branches, along with a dual label assignment strategy designed to enable NMS-free inference. Unlike conventional object detectors that rely on non-maximum suppression (NMS) as a post-processing step, YOLOv10 removes this step entirely by applying one-to-one label assignment during training. This consistent label allocation aligns training and inference stages, reduces prediction redundancy, and enables fast, end-to-end deployment suitable for clinical integration. In addition, the Rank-based Anchor-Free Head introduced in YOLOv10 further improves target box ranking by separating confidence scoring from box localization, thus improving the precision of final predictions.

The training parameters included the use of the Stochastic Gradient Descent (SGD) optimizer, with an initial learning rate of 0.01 that linearly decayed to 0.0001 over the course of 80 training epochs. Data augmentation techniques such as Mosaic, Mixup, Copy-paste, Flipping, and Rotation were also applied during the training process.

### 2.5. Performance Evaluation

To evaluate the model’s performance, the following metrics were used, as they have been shown in previous studies to be appropriate for assessing YOLO-based models [33]:

Mean Average Precision (mAP). mAP is a comprehensive metric for assessing the performance of object detection models. It calculates the area under the precision-recall curve for each predicted box to obtain the Average Precision (AP), and then takes the mean across all classes. The formula is as follows:

(1) $$mAP=\frac{1}{N}\sum_{i=1}^{N}{AP}_{i}$$

where N represents the total number of classes, i denotes the i-th class, and APi is the Average Precision of the i-th class.

Recall. Measures the proportion of actual lesions detected by the model. The formula is as follows:

(2) $$Recall=\frac{TP}{TP+FN}$$

where TP (True Positives) are correctly identified lesions, and FN (False Negatives) are actual lesions missed by the model.

Precision. Measures the proportion of predicted lesions that are actual lesions. The formula is as follows:

(3) $$Precision=\frac{TP}{TP+FP}$$

where FP (False Positives) are instances incorrectly identified as lesions by the model.

During the training process, these metrics were continuously monitored, and model parameters were adjusted accordingly to achieve optimal performance.

## 3. Results

The study included a total of 31 patients with NSTIs, with a mean age of 62.13 years. Of the patients, 22 were male and 9 were female (Table 1). Diabetes was the most common underlying condition, present in 64.52% of patients. Microbiological cultures revealed that *Klebsiella pneumoniae* was the most frequently isolated pathogen among the NSTI cases, with polymicrobial infections being common. The median time from CT image acquisition to final radiology report was 10.08 h. The distribution of NSTI involvement by body region was as follows: the lower extremities were most affected (41.54%), followed by the perineum (38.97%), upper extremities, head and neck, and chest and abdominal wall.

A total of 9001 images were analyzed, including 3332 NSTI images and 5669 healthy images. These were divided into a training dataset of 6306 images, a validation data set of 1799 images, and a test dataset of 896 images. The CT imaging features of NSTI were further categorized into four major findings: soft tissue ectopic gas was the most prevalent feature (33.13%), followed by fascia edematous changes (32.10%), fluid accumulation (27.04%), and soft tissue non-enhancement (6.88%) (Table 2).

**Figure 5:**
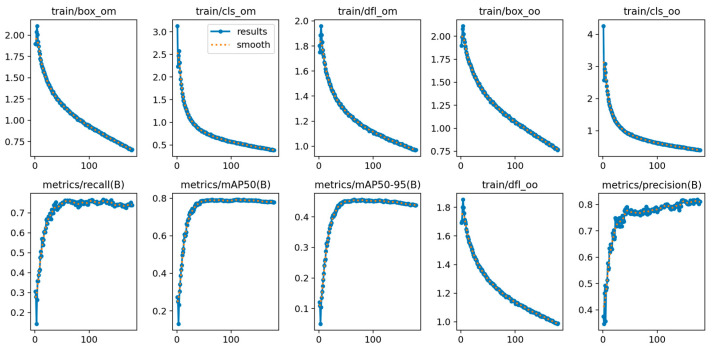
Model performance trends across iterations. Training and validation metrics include bounding box loss, class loss, distribution loss, precision, recall, mAP at 50, and mAP at 95.

The main performance metrics of the model on the test set include a mAP of 0.75, a recall of 0.74, and a precision of 0.72. Figure 5 reveals the trends of model performance metrics with each iteration. The subplots from the top left to the right represent the one-to-many losses for bounding boxes, classification, and bounding box distribution, followed by the one-to-one losses for bounding boxes and classification. In the lower section, the subplots from left to right show recall, mAP at 50, mAP at 50–95, the one-to-one loss for bounding box distribution, and precision. The two mAP metrics represent the mAP performance under different thresholds. Among the various losses, it can be observed that they gradually decrease with each epoch, reaching their lowest point towards the end of training. For each evaluation metric, they increase progressively with each epoch and stabilize at a high level by the end of training.

**Figure 6:**
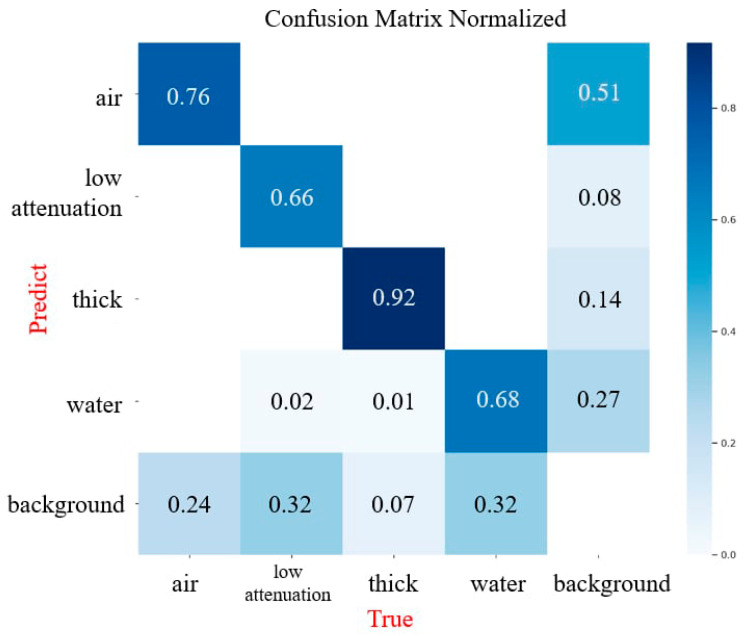
Normalized confusion matrix illustrating the model’s classification performance for each NSTI feature and the background class.

The normalized confusion matrix provides detailed insights into the detection performance for each NSTI feature and the background class (Figure 6). The recall for soft tissue ectopic gas (“air”) is 0.76, with a notable false positive rate of 0.51. Soft tissue non-enhancement (“low attenuation”) achieves a recall of 0.66, with 0.32 of the background being misclassified. Fascia edematous change (“thick”) shows the highest recall at 0.92, with minimal confusion across other categories. Fluid accumulation (“water”) has a recall of 0.68, with 0.27 of the background being misclassified as fluid accumulation.

**Figure 7:**
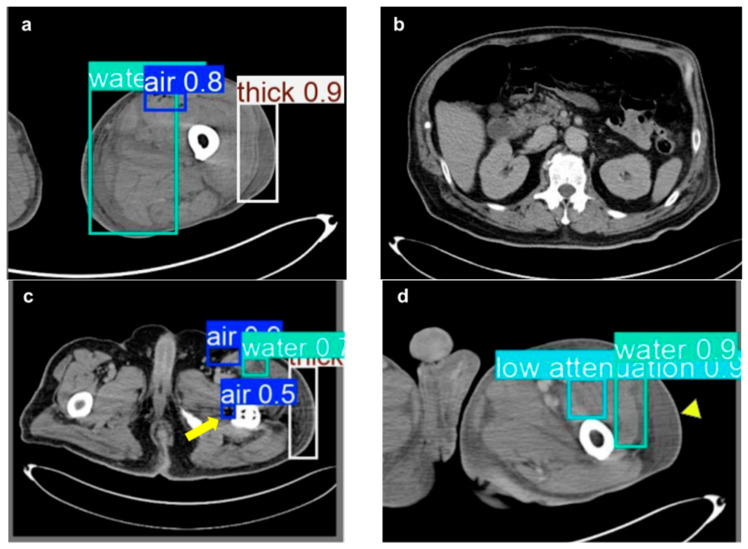
Detection examples visualizing the model’s performance on test set CT images. (**a**) Multiple features correctly detected in the NSTI image. (**b**) No NSTI features found in healthy images. (**c**) Implant shadow misclassified (yellow arrow). (**d**) Missed fascia edematous change (yellow arrowhead).

Figure 7 provides an overview of the model’s performance in detecting NSTI features on CT images from the test set, where each identified feature is marked with a bounding box and its corresponding confidence score is displayed in the upper right corner. In Figure 7a, the model accurately detects multiple NSTI features, including soft tissue ectopic gas and fluid accumulation in the subfascial and intermuscular planes, as well as fascia edematous changes in the lateral thigh, demonstrating its ability to identify distinct NSTI features. Figure 7b presents a healthy CT image, where the model appropriately did not detect any NSTI features, indicating no false positives in this instance. In Figure 7c, the yellow arrow marks an error in which the model mistakenly classified an implant-related shadow as soft tissue ectopic gas. In Figure 7d, the yellow arrowhead highlights a region of fascia edematous change that the model did not identify, reflecting a missed feature in the annotated area.

## 4. Discussion

This study focuses on developing an automated system utilizing the YOLOv10 model to identify NSTI features in CT images. Using a dataset of 9001 annotated CT images, the model demonstrated exceptional performance during both training and testing. It achieved a mAP of 0.75 on the test set, highlighting its accuracy in identifying NSTI features such as soft tissue ectopic gas, fascia edematous change, soft tissue non-enhancement, and fluid accumulation. With a recall of 0.74 and a precision of 0.72, the model effectively detected most actual lesions while minimizing false positives. Additionally, the YOLOv10 model’s emphasis on speed and efficiency makes it highly suitable for real-time clinical applications requiring timely diagnosis in emergency settings [34]. These results illustrate the potential of the YOLOv10 model to enhance automated NSTI detection in clinical settings, assisting radiologists in quickly identifying NSTI-affected areas within a CT series and aiding surgeons in formulating more detailed and precise surgical plans.

We identified four key radiologic features characteristic of NSTI: soft tissue ectopic gas, fluid accumulation, fascial edematous changes, and soft tissue non-enhancement [26]. The detection of soft tissues ectopic gas, particularly along fascial planes, is a well-established radiologic hallmark of NSTIs, most associated with prolonged anaerobic infection [16,21,22,23]. This imaging feature is notably absent in less aggressive soft tissue conditions such as cellulitis, enhancing its diagnostic specificity. Among the various radiologic signs, soft tissue gas is especially indicative of type I polymicrobial necrotizing fasciitis and serves as an early and actionable clue. Its presence on CT images or conventional radiographs should prompt expedited surgical assessment to minimize diagnostic delays and reduce associated morbidity [1]. Although the presence of soft tissue ectopic gas along fascial planes is a specific radiologic sign of NSTI, its absence—particularly in early disease stages or in diabetic patients—does not exclude the diagnosis [11]. Fluid accumulation within the subfascial plane and intermuscular septum, or abscess are key imaging features in the early diagnosis of NSTIs. On CT, these collections often appear before gas formation, particularly during the early stages of the disease. They typically present as subfascial or intermuscular fluid with attenuation values exceeding 10 Hounsfield units (HU) [11,24,25,26]. This finding, especially when combined with fascial thickening, enhances diagnostic sensitivity and helps distinguish NSTIs from less severe soft tissue infections such as cellulitis or non-necrotizing fasciitis [11,29]. Several studies have identified soft tissue non-enhancement on contrast-enhanced CT as a potentially valuable indicator of necrosis in necrotizing fasciitis. The lack of fascial enhancement is a key radiologic marker that distinguishes necrotizing from non-necrotizing fasciitis [31]. Carbonetti et al. incorporated non-enhancement of the muscular fascia into their CT-based scoring system, reporting it in 92% of NF cases and noting a strong correlation with confirmed necrosis, though acknowledging its imperfect specificity [32]. The review by Ali et al. also supported this feature, noting that absence of enhancement in deep fascia or muscle may suggest necrosis but could also result from severe edema or hypoperfusion, warranting cautious interpretation [23]. Fascial edematous change is widely recognized as a key imaging feature of NSTIs, typically presenting as fascial thickening greater than 3 mm accompanied by inflammatory signs such as fat stranding, and in some cases, lack of fascial enhancement suggestive of necrosis [11,23,27,28,29,30]. It is considered one of the earliest and most indicative signs on CT. Unlike the diffuse, symmetric edema seen in non-infectious conditions such as heart failure, fascial thickening in NSTIs typically shows a localized, asymmetric distribution that corresponds to the affected compartment. However, similar findings may also be observed in cases of severe cellulitis, limiting its specificity [23,29,30]. These features were incorporated into the CT-based scoring system for NSTI proposed by McGillicuddy et al., with reported odds ratios of 22.6 for soft tissue gas, 6.3 for fascial edematous changes, and 2.6 for fluid accumulation [16].

We achieved high recall rates for the features of soft tissue ectopic gas and fascial edematous change, at 0.76 and 0.92, respectively. The main issue in the prediction of soft tissue gas was a high false positive rate of 0.51. Visualization revealed that these erroneous predictions were due to the model misidentifying relatively low-density areas within the soft tissue, such as subcutaneous fat, as soft tissue gas. In fact, the model did not misclassify gas present in normal anatomical structures, such as the aerodigestive tract or paranasal sinuses. The recall for fluid accumulation was only 0.66, possibly because fluid is typically found in the intermuscular septum, where it appears irregular and varies in shape across different cross-sectional images. The object detection model used in this study struggles to achieve the same level of performance in detecting features with varying shapes as it does for those with consistent shapes. The performance of soft tissue non-enhancement in the model was inferior compared to other features, which may be attributed to the insufficient numbers of this feature in the dataset. Although the model cannot predict every feature, these features in NSTI images typically appear in a continuous pattern. By detecting only part of the lesions, the model can still significantly reduce the time required for radiologists or surgeons to make the diagnosis.

Artificial intelligence is extensively utilized in musculoskeletal radiology for tasks such as classifying soft tissue tumors as benign or malignant [35,36], delineating lesion boundaries [37], and detecting high-grade soft tissue sarcomas [38] or chest wall abnormalities [39]. We employed the YOLOv10 model as the pre-trained model for this study. YOLOv10 represents a significant advancement in the YOLO model series, aimed at further improving detection accuracy and computational efficiency. The YOLO model series are popular for their good balance between computational cost and detection performance [40], widely used in various practical scenarios such as autonomous driving [41], robot navigation [42], and object tracking [43]. Building upon its predecessors, YOLOv10 introduces architectural and training enhancements that streamline the detection pipeline. First, it eliminates the traditional reliance on NMS during inference, reducing latency and enabling more efficient end-to-end deployment. In addition, modifications to the model architecture—such as optimized classification heads and downsampling mechanisms—help lower computational overheads while maintaining or improving accuracy. These refinements make YOLOv10 a highly effective solution for real-time object detection tasks. Currently, few studies have reported the application of YOLOv10 in medical imaging [44,45,46]. YOLOv10 has demonstrated superior performance and efficiency across multiple benchmark datasets. For instance, compared to YOLOv9-C, YOLOv10-B reduces latency by 46%, decreases the number of parameters by 25%, and maintains comparable performance [47,48]. These improvements establish YOLOv10 as a leading real-time object detection model, balancing high accuracy with low computational cost, making it suitable for diverse real-time applications. In addition, the dataset used in this study contained more healthy images than NSTI images, resulting in a mild class imbalance. However, the annotations were based on localized lesion features rather than overall image-level classifications. Since the YOLO model is specifically designed for object detection tasks that emphasize local feature identification, its performance is less affected by class distribution, making it well-suited to the structure and objectives of our dataset.

A compelling demonstration of the effectiveness of YOLOv10 is reflected in the results of our study, which achieved an approximately 20% improvement in mAP compared to the 2021 study by Das et al., which focused on detecting the external appearance of NSTI [20]. In a more recent study, Sheeram et al. proposed a YOLOv9-based model utilizing clinical photographs of affected skin regions, achieving an Intersection over Union (IoU) of 0.649, thereby demonstrating its potential for recognizing superficial manifestations of necrotizing fasciitis [49]. In contrast, our YOLOv10-based model attained a markedly higher detection performance (mAP 0.75), leveraging CT images to identify detailed radiologic features of NSTI. This improvement may be attributed to both the superior anatomical resolution provided by CT and the architectural enhancements of the YOLOv10 framework. Furthermore, by explicitly annotating key radiologic features, this study enables AI-driven outputs to provide localized, feature-specific predictions directly overlaid on CT images. This may assist clinicians in rapidly identifying the location and nature of potential lesions, reducing the need to manually search for abnormalities based solely on a general classification result. Compared to conventional black-box classification models, this approach offers more clinically actionable and targeted information to support timely diagnosis and surgical planning. Table 3 summarizes key information from the three studies that applied deep learning techniques for the detection of necrotizing soft tissue infections.

It is essential to emphasize that the findings of this study cannot serve as a definitive diagnostic tool for NSTI. Instead, we aim to leverage the integration of AI models to help surgeons more accurately delineate the surgical range on CT images and to alleviate the workload of radiologists in interpreting emergency radiographic imaging. All the imaging data in this study were sourced exclusively from Chi Mei Medical Center, with no external institutional data, resulting in a lack of external validity. Our imaging data were obtained from different CT machines, and we annotated specific features rather than the disease itself, which may mitigate the impact of limited external validity. This study did not include other soft tissue imaging, such as cellulitis, meaning the model’s performance on other soft tissue infections remains unverified and requires further investigation. Moreover, since NSTI commonly affects the perineum region and lower extremities [50], the distribution of body parts in our dataset is not perfectly balanced. Further case collection is required in the future to include more images from other regions, thereby enhancing the generalizability of our model.

This study is, to the best of our knowledge, the first to apply artificial intelligence to assist in the detection of soft tissue infections on CT images, introducing a novel application scenario for AI in soft tissue imaging. A preliminary version of this work was previously presented [51], where the proposed model demonstrated promising performance and received positive feedback from attendees regarding its potential clinical utility. By adopting a feature-based detection approach rather than simple binary classification, the proposed model enables visual localization and labeling of clinically meaningful features. This enhances clinical explainability and facilitates clinical decision-making. In future work, the model will first undergo further refinement and rigorous validation to ensure its safety and clinical reliability. Once these steps are completed, it will be integrated into clinical viewing systems, where prospective evaluations will be conducted to collect structured feedback from radiologists, surgeons, and emergency physicians regarding its usability and diagnostic utility. These efforts aim to further optimize the model’s interface and assess its practical value in real-world clinical settings.

## 5. Conclusions

In conclusion, this study highlights the potential of artificial intelligence in detecting NSTI features on CT images, demonstrating its capability in identifying key radiologic markers. The model could be beneficial for real-time clinical applications, assisting radiologists and surgeons in diagnosis and surgical planning while reducing their diagnostic workload. By applying AI to the diagnosis of soft tissue infections, this study presents a novel approach to integrating advanced technologies into clinical practice, laying the foundation for further research and expanded applications in medical imaging.

## Figures and Tables

**Table 1:** Summary of the patients’ demographics characteristics ^1^.

Characteristic	Number (Mean)	Percentage (SD)
Gender		
Male	22	70.97%
Female	9	29.03%
Age		
	62.13	13.56
Admission source		
Emergency	27	87.10%
Inpatient	3	9.68%
Outpatient	1	3.23%
Comorbidity		
Diabetes	20	64.52%
Peripheral vascular disease	1	3.23%
Trauma at the affected site	4	12.90%
Cirrhosis	4	12.90%
End stage renal disease	4	12.90%
Chemotherapy	2	6.45%
Hospitalization		
General ward	17	54.84%
Intensive care unit	14	45.16%
Expire in 48 h	2	6.45%
Causative pathogens		
Monomicrobial	16	51.61%
*Staphylococcus aureus (MRSA)*	2	
*Staphylococcus capitis*	1	
*Coagulase negative staphylococcus*	1	
*Enterococcus faecalis*	1	
*Klebsiella pneumoniae*	6	
*Vibrio vulnificus*	2	
*Vibrio parahaemolyticus*	1	
*Enterobacter cloacae*	1	
*Enterobacter bugandensis*	1	
Polymicrobial	12	38.71%
Mixed aerobic	9	
Mixed aerobic/anaerobic	3	
Mixed bacteria/fungus	1	
Culture negative	3	9.68%
NSTI-affected region		
Head and neck	2	6.45%
Upper extremities	3	9.68%
Chest and abdominal wall	2	6.45%
Perineum	10	32.26%
Lower extremities	14	45.16%
Time from admission to surgery (day)		
	2.53	6.07
Time from CT scan to report (hour)		
	10.08	11.79

^1^ Continuous variables are presented as mean [standard deviation (SD)], while categorical variables are expressed as number (percentage).

**Table 2:** Summary of the CT imaging features.

Category	Number	Percentage
NSTI-affected regions		
Head and neck	427	4.42%
Upper extremities	598	6.19%
Chest and abdominal wall	859	8.89%
Perineum	3767	38.97%
Lower extremities	4016	41.54%
Lesion images		
NSTI images	3332	37.02%
Health images	5669	67.98%
NSTI features		
Soft tissue ectopic gas	1982	33.13%
Fluid accumulation	1577	27.04%
Fascia edematous changes	1872	32.10%
Soft tissue non-enhancement	401	6.88%

**Table 3:** Overview of deep learning approaches for NSTI detection across imaging modalities.

Authors, Year	Study Design	Dataset	Model	Target Features	Performance
Das et al., 2021 [20]	Retrospective study	693 images in total; 231 Clinical skin images containing NSTI; 231 Clinical skin images containing normal skin; 231 Augmented images containing NSTI	YOLOv3	Skin appearance suggestive of NSTI	AP: 0.58
P. Shreeram et al., 2025 [49]	Retrospective study	693 images in total; 231 Clinical skin images containing NSTI; 231 Clinical skin images containing normal skin; 231 Augmented images containing NSTI	YOLOv9	Skin appearance suggestive of NSTI	IoU: 0.649
Heng-Yu Lin et al., 2025	Retrospective study	9001 CT images in total; 3332 NSTI images and 5669 healthy images.	YOLOv10	Soft tissue ectopic gas, fluid accumulation, fascia edematous changes, soft tissue non-enhancement in CT images	mAP: 0.75

AP, Average Precision; IoU, Intersection over Union.

## Data Availability

Data available on request due to restrictions (e.g., privacy, legal, or ethical reasons).

## Abbreviations

The following abbreviations are used in this manuscript:

NSTI	Necrotizing Soft Tissue Infection
CT	Computed Tomography
Yolov10	You Only Look Once version 10
mAP	Mean Average Precision
LRINEC	Laboratory Risk Indicator for Necrotizing Fasciitis
MRI	Magnetic Resonance Imaging
CSPNet	Cross-Stage Partial Network
SCD	Spatial-Channel Decoupled Downsampling
PAN	Path Aggregation Network
NMS	Non-Maximum Suppression
SGD	Stochastic Gradient Descent
AP	Average Precision
TP	True Positive
FN	False Negative
FP	False Positive
SD	Standard deviation
IoU	Intersection over Union

## References

[1] Stevens D.L., Bryant A.E. (2017). Necrotizing Soft-Tissue Infections. N. Engl. J. Med..

[2] Tedesco S., Di Grezia M., Tropeano G., Altieri G., Brisinda G. (2025). Necrotizing soft tissue infections: A surgical narrative review. Updates Surg..

[3] Bisgaard E.K., Bulger E.M. (2023). Current diagnosis and management of necrotizing soft tissue infections: What you need to know. J. Trauma Acute Care Surg..

[4] Naseer U., Steinbakk M., Blystad H., Caugant D. (2016). Epidemiology of invasive group A streptococcal infections in Norway 2010–2014: A retrospective cohort study. Eur. J. Clin. Microbiol. Infect. Dis..

[5] Hakkarainen T.W., Kopari N.M., Pham T.N., Evans H.L. (2014). Necrotizing soft tissue infections: Review and current concepts in treatment, systems of care, and outcomes. Curr. Probl. Surg..

[6] Lancerotto L., Tocco I., Salmaso R., Vindigni V., Bassetto F. (2012). Necrotizing fasciitis: Classification, diagnosis, and management. J. Trauma Acute Care Surg..

[7] Fernando S.M., Tran A., Cheng W., Rochwerg B., Kyeremanteng K., Seely A.J.E., Inaba K., Perry J.J. (2019). Necrotizing Soft Tissue Infection: Diagnostic Accuracy of Physical Examination, Imaging, and LRINEC Score: A Systematic Review and Meta-Analysis. Ann. Surg..

[8] Wei X.K., Huo J.Y., Yang Q., Li J. (2024). Early diagnosis of necrotizing fasciitis: Imaging techniques and their combined application. Int. Wound J..

[9] Marks A., Patel D., Sundaram T., Johnson J., Gottlieb M. (2023). Ultrasound for the diagnosis of necrotizing fasciitis: A systematic review of the literature. Am. J. Emerg. Med..

[10] Clark M.L., Fisher K.L. (2017). Sonographic detection of necrotizing fasciitis. J. Diagn. Med. Sonogr..

[11] Tso D.K., Singh A.K. (2018). Necrotizing fasciitis of the lower extremity: Imaging pearls and pitfalls. Br. J. Radiol..

[12] Lin C.-N., Hsiao C.-T., Chang C.-P., Huang T.-Y., Hsiao K.-Y., Chen Y.-C., Fann W.-C. (2019). The relationship between fluid accumulation in ultrasonography and the diagnosis and prognosis of patients with necrotizing fasciitis. Ultrasound Med. Biol..

[13] Gan R.K., Sanchez Martinez A., Abu Hasan M.A.-S., Castro Delgado R., Arcos González P. (2023). Point-of-care ultrasonography in diagnosing necrotizing fasciitis—A literature review. J. Ultrasound.

[14] Chaudhry A.A., Baker K.S., Gould E.S., Gupta R. (2015). Necrotizing fasciitis and its mimics: What radiologists need to know. AJR Am. J. Roentgenol..

[15] Turecki M.B., Taljanovic M.S., Stubbs A.Y., Graham A.R., Holden D.A., Hunter T.B., Rogers L.F. (2010). Imaging of musculoskeletal soft tissue infections. Skelet. Radiol..

[16] McGillicuddy E.A., Lischuk A.W., Schuster K.M., Kaplan L.J., Maung A., Lui F.Y., Bokhari S.A., Davis K.A. (2011). Development of a computed tomography-based scoring system for necrotizing soft-tissue infections. J. Trauma.

[17] Hames K., Patlas M.N., Mellnick V.M., Katz D.S. (2019). Errors in emergency and trauma radiology: General principles. Errors Emerg. Trauma Radiol..

[18] Katzman B.D., van der Pol C.B., Soyer P., Patlas M.N. (2023). Artificial intelligence in emergency radiology: A review of applications and possibilities. Diagn. Interv. Imaging.

[19] Chiu M.-C., Tseng T.-Y., Chang C.-C., Chen C.-C. (2023). Applying Deep Learning to Establish a Telemedicine Assistance System: A Case Study of the Stage Classification of Pressure Injuries. Leveraging Transdisciplinary Engineering in a Changing and Connected World.

[20] Das A., Amin S., Hughes J.A. Automatic Detection of Necrotizing Fasciitis: A Dataset and Early Results. Proceedings of the 2021 IEEE Conference on Computational Intelligence in Bioinformatics and Computational Biology (CIBCB).

[21] Leturia Etxeberria M., Biurrun Mancisidor M.C., Ugarte Nuño A., Arenaza Choperena G., Mendoza Alonso M., Esnaola Albizu M., Serdio Mier A., Gredilla Sáenz M., Gomez Usabiaga V. (2020). Imaging Assessment of Ectopic Gas Collections. Radiographics.

[22] Chingkoe C.M., Jahed A., Loreto M.P., Sarrazin J., McGregor C.T., Blaichman J.I., Glanc P. (2015). Retroperitoneal fasciitis: Spectrum of CT findings in the abdomen and pelvis. Radiographics.

[23] Hayeri M.R., Ziai P., Shehata M.L., Teytelboym O.M., Huang B.K. (2016). Soft-Tissue Infections and Their Imaging Mimics: From Cellulitis to Necrotizing Fasciitis. Radiographics.

[24] Bruls R.J., Kwee R.M. (2021). CT in necrotizing soft tissue infection: Diagnostic criteria and comparison with LRINEC score. Eur. Radiol..

[25] Meyer H.-J., Schnarkowski B., Leonhardi J., Mehdorn M., Ebel S., Goessmann H., Denecke T. (2021). CT Texture analysis and CT scores for characterization of fluid collections. BMC Med. Imaging.

[26] Skusa C., Skusa R., Wohlfarth M., Warnke P., Podbielski A., Bath K., Groß J., Schafmayer C., Frickmann H., Weber M.-A. (2022). Imaging and clinical parameters for distinction between infected and non-infected fluid collections in CT: Prospective study using extended microbiological approach. Diagnostics.

[27] Spinnato P., Patel D.B., Di Carlo M., Bartoloni A., Cevolani L., Matcuk G.R., Crombé A. (2022). Imaging of musculoskeletal soft-tissue infections in clinical practice: A comprehensive updated review. Microorganisms.

[28] Paz Maya S., Dualde Beltrán D., Lemercier P., Leiva-Salinas C. (2014). Necrotizing fasciitis: An urgent diagnosis. Skelet. Radiol..

[29] Kolinsky D.C., Liang S.Y. (2018). Musculoskeletal Infections in the Emergency Department. Emerg. Med. Clin. N. Am..

[30] Wysoki M.G., Santora T.A., Shah R.M., Friedman A.C. (1997). Necrotizing fasciitis: CT characteristics. Radiology.

[31] Fayad L.M., Carrino J.A., Fishman E.K. (2007). Musculoskeletal infection: Role of CT in the emergency department. Radiographics.

[32] Carbonetti F., Cremona A., Carusi V., Guidi M., Iannicelli E., Di Girolamo M., Sergi D., Clarioni A., Baio G., Antonelli G. (2016). The role of contrast enhanced computed tomography in the diagnosis of necrotizing fasciitis and comparison with the laboratory risk indicator for necrotizing fasciitis (LRINEC). Radiol. Med..

[33] Redmon J., Divvala S., Girshick R., Farhadi A. You only look once: Unified, real-time object detection. Proceedings of the IEEE Conference on Computer Vision and Pattern Recognition.

[34] Yeerjiang A., Wang Z., Huang X., Zhang J., Chen Q., Qin Y., He J. (2024). YOLOv1 to YOLOv10: A Comprehensive Review of YOLO Variants and Their Application in Medical Image Detection. J. Artif. Intell. Pract..

[35] Crombé A., Spinnato P., Italiano A., Brisse H.J., Feydy A., Fadli D., Kind M. (2023). Radiomics and artificial intelligence for soft-tissue sarcomas: Current status and perspectives. Diagn. Interv. Imaging.

[36] Gitto S., Interlenghi M., Cuocolo R., Salvatore C., Giannetta V., Badalyan J., Gallazzi E., Spinelli M.S., Gallazzi M., Serpi F. (2023). MRI radiomics-based machine learning for classification of deep-seated lipoma and atypical lipomatous tumor of the extremities. La Radiol. Medica.

[37] Wang S., Sun M., Sun J., Wang Q., Wang G., Wang X., Meng X., Wang Z., Yu H. (2024). Advancing musculoskeletal tumor diagnosis: Automated segmentation and predictive classification using deep learning and radiomics. Comput. Biol. Med..

[38] Yang Y., Zhou Y., Zhou C., Zhang X., Ma X. (2022). MRI-based computer-aided diagnostic model to predict tumor grading and clinical outcomes in patients with soft tissue sarcoma. J. Magn. Reson. Imaging.

[39] Chiu M.-C., Tsai S.C.-S., Bai Z.-R., Lin A., Chang C.-C., Wang G.-Z., Lin F.C.-F. (2024). Radiographic chest wall abnormalities in primary spontaneous pneumothorax identified by artificial intelligence. Heliyon.

[40] Jiang P., Ergu D., Liu F., Cai Y., Ma B. (2022). A Review of Yolo algorithm developments. Procedia Comput. Sci..

[41] Huu P.N., Pham Thi Q., Tong Thi Quynh P. (2022). Proposing Lane and Obstacle Detection Algorithm Using YOLO to Control Self-Driving Cars on Advanced Networks. Adv. Multimed..

[42] Dos Reis D.H., Welfer D., De Souza Leite Cuadros M.A., Gamarra D.F.T. (2019). Mobile robot navigation using an object recognition software with RGBD images and the YOLO algorithm. Appl. Artif. Intell..

[43] Zheng Z., Li J., Qin L. (2023). YOLO-BYTE: An efficient multi-object tracking algorithm for automatic monitoring of dairy cows. Comput. Electron. Agric..

[44] Byeon H. (2024). YOLO v10-Based Brain Tumor Detection: An Innovative Approach in CT Imaging. Nanotechnol. Percept..

[45] Srinivasu P.N., Kumari G.L.A., Narahari S.C., Ahmed S., Alhumam A. (2025). Exploring the impact of hyperparameter and data augmentation in YOLO V10 for accurate bone fracture detection from X-ray images. Sci. Rep..

[46] Mahapadi A.A., Shirsath V., Pundge A. (2025). Real-Time Diabetic Retinopathy Detection Using YOLO-v10 with Nature-Inspired Optimization. Biomed. Mater. Devices.

[47] Wang A., Chen H., Liu L., Chen K., Lin Z., Han J., Ding G. (2024). Yolov10: Real-time end-to-end object detection. arXiv.

[48] Sapkota R., Qureshi R., Calero M.F., Hussain M., Badjugar C., Nepal U., Poulose A., Zeno P., Vaddevolu U.B.P., Yan H. (2024). Yolov10 to its genesis: A decadal and comprehensive review of the you only look once series. arXiv.

[49] Shreeram P., Krithik P., Sountharrajan S., Saranya S. (2025). Automated Detection of Necrotizing Fasciitis in Patient Affected Area Images using YOLO v9. Curr. Sci..

[50] Becker M., Zbären P., Hermans R., Becker C.D., Marchal F., Kurt A.-M., Marre S., Rüfenacht D., Terrier F. (1997). Necrotizing fasciitis of the head and neck: Role of CT in diagnosis and management. Radiology.

[51] Kao T.-L., Chiu M.-C., Chen C.-C., Lin H.-Y. Develop a Real Time Necrotizing Fasciitis Identification Model to Enhance Clinical Diagnosis Efficiency and Accuracy in Computed Tomography Images. Proceedings of the Asia Pacific Industrial Engineering & Management Systems Conference 2024.

